# Neglected Tropical Diseases in the Ebola-Affected Countries of West Africa

**DOI:** 10.1371/journal.pntd.0003671

**Published:** 2015-06-25

**Authors:** Peter J. Hotez

**Affiliations:** 1 Sabin Vaccine Institute and Texas Children’s Hospital Center for Vaccine Development, Department of Pediatrics and Molecular Virology and Microbiology, National School of Tropical Medicine, Baylor College of Medicine, Houston, Texas, United States of America; 2 Department of Biology, Baylor University, Waco, Texas, United States of America; 3 James A. Baker III Institute for Public Policy, Rice University, Houston, Texas, United States of America; University of Washington Medical Center, UNITED STATES

While global attention in West Africa is focused on the emergence of Ebola virus infection, new information from the published literature and World Health Organization databases reveals that many other neglected tropical diseases (NTDs) are far more widespread and also require urgent attention.

**Fig 1:**
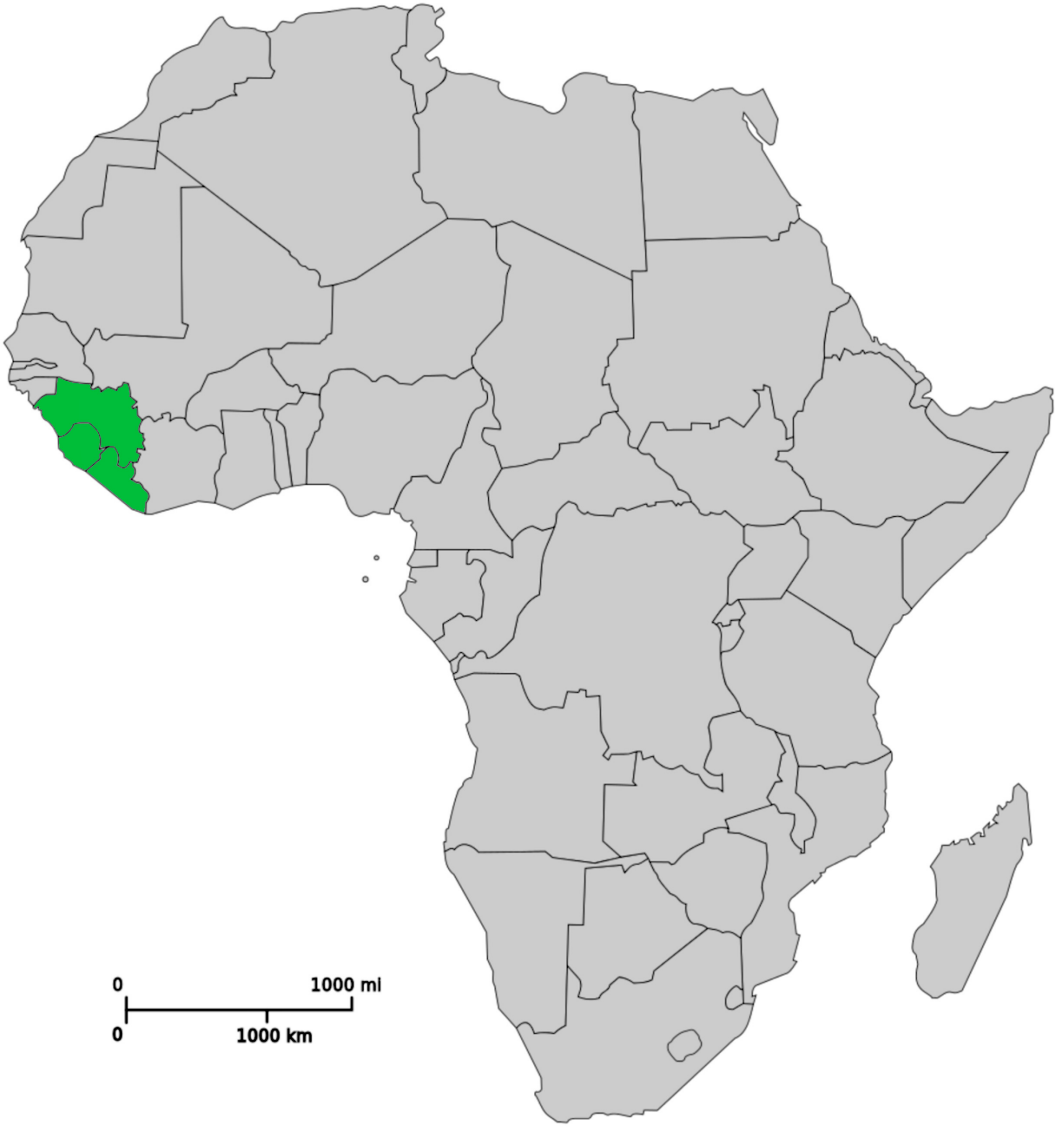
Highlighted in green, north to south: Guinea, Sierra Leone, and Liberia, the Ebola-affected Countries of West Africa. Modified from public-domain image accessed through Wikipedia: http://commons.wikimedia.org/wiki/File:Blank_Map-Africa.svg.

Well before Ebola virus infection emerged in West Africa at the end of 2013, the three major affected countries—Guinea, Liberia, and Sierra Leone (Fig 1) – were already known to be highly affected by NTDs. Sierra Leone, in particular, as a part of British West Africa for almost two centuries, was a well-established destination for scientific investigation on these diseases. The British physician (and slave abolitionist) Dr. Thomas Masterman Winterbottom first identified the characteristic posterior cervical lymphadenopathy linked to human African trypanosomiasis (HAT) while working in Sierra Leone in the early 1800s [1]. Later, with the establishment of the Liverpool School of Tropical Medicine at the turn of the 20th century, extensive research activities were conducted on the epidemiology of HAT and other NTDs in Sierra Leone [2], and the Alfred Jones Research Laboratory was established in Freetown, where the transmission of onchocerciasis by black flies was discovered by Blacklock and his colleagues [3].

Today, new information from the recent literature and the World Health Organization (WHO) Preventive Chemotherapy and Transmission (PCT) Control databases reveals that NTDs remain widespread in Guinea, Liberia, and Sierra Leone (Table 1) [4–11]. An estimated 22.6 million people live in these three nations, where (as of this writing) approximately 27,000 have been infected with the Ebola virus, resulting in almost 9,000 deaths [5]. However, other NTDs now affect millions of people. According to a new estimate led by the Swiss Tropical and Public Health Institute, more than 2 million children suffer from hookworm infection in the three affected countries, and Sierra Leone has the highest prevalence of hookworm infection in sub-Saharan Africa [6]. Almost 700,000 people are also infected with ascariasis and trichuriasis [6]. Schistosomiasis is equally or even more widespread, as almost 5 million people require preventive chemotherapy (PC) with praziquantel [7]. Approximately 100,000 require PC for lymphatic filariasis [8], while more than 40% of the combined populations of the three countries are at risk for onchocerciasis [9].

**Table 1:** The neglected tropical diseases in the three Ebola-affected countries of West Africa.

Disease or Category	Guinea	Liberia	Sierra Leone	All three countries	Reference
Population	12.0 million	4.4 million	6.2 million	22.6 million	[4]
Ebola virus infection (as of June 1, 2015)	3,653	12,816	10,666	27,135	[5]
Hookworm^1^ (Children)	0.99 million	0.31 million	0.84 million	2.14 million	[6]
Ascariasis^1^ (Children)	0.17 million	0.12 million	0.15 million	0.44 million	[6]
Trichuriasis^1^ (Children)	0.08 million	0.09 million	0.06 million	0.23 million	[6]
Schistosomiasis (Population requiring Rx in 2013)	2.11 million	1.07 million	1.47 million	4.65 million	[7]
Lymphatic Filariasis (Proportion of population requiring preventive chemotherapy in 2013)	0.06 million 0.4885%	0.01 million 0.2366%	0.03 million 0.5492%	0.10 million	[8]
Onchocerciasis (Total population at risk targeted [estimated] in 2013)	3.28 million	3.09 million	3.18 million	9.55 million	[9]
Malaria (Reported or confirmed cases)	0.21 million	1.24 million	1.70 million	3.15 million	[10]
Dengue (Apparent cases)	0.19 million	0.10 million	0.14 million	0.43 million	[11]

^1^Final numbers obtained by multiplying the prevalence estimates by the pediatric population estimates provided in [65].

Malaria remains widespread. According to the WHO, there are more than 3 million reported cases, almost all of which are caused by *Plasmodium falciparum* [10]. But in addition, hookworm and falciparum malaria co-infections leading to severe anemia and morbidity are also widespread [12], as are schistosomiasis and falciparum malaria co-infections [13]. Dengue fever has emerged with almost half a million “apparent” cases, in addition to many more inapparent cases [11].

In total, at least 10 million people in Guinea, Liberia, and Sierra Leone (almost one-half of the total population) suffer from at least one NTD and/or malaria, and much of the population suffers simultaneously from several NTDs, especially the intestinal helminth infections, schistosomiasis, and malaria. In response, the United States Agency for International Development (USAID) has provided large-scale support to launch national programs of NTD control to simultaneously target the intestinal helminth infections, schistosomiasis, lymphatic filariasis, onchocerciasis, and trachoma with a package of several essential NTD medicines [14]. According to USAID, the entire population of Guinea is at risk for these NTDs [15]. They began support in 2011, and mass treatment programs for trachoma and lymphatic filariasis (LF) subsequently began in 2012 and 2013, respectively [15]. Since 2012, more than 2 million treatments have been provided to more than 1 million people in Guinea [15]. Similarly, USAID support has enabled Sierra Leone’s NTD program to provide an estimated 57 million NTD treatments to approximately 22 million people, with a track record of success in providing treatments in complicated, highly populated, urban settings [16]. In Liberia in 2012, a Liberian National NTD Master Plan was created, which works with several public–private partnerships and the United Kingdom Department of International Development (DFID), together with the private Ending Neglected Diseases (END) Fund [17].

The Global Fund to Fight AIDS, Tuberculosis, and Malaria (GFATM) also supports malaria control efforts in Guinea, Liberia, and Sierra Leone, where an estimated 4.5 million, 2.8 million, and 0.8 million bed nets have been distributed, respectively [18–20]. The US President’s Malaria Initiative (PMI) is also providing extensive financial and logistical support in Guinea and Liberia [21,22].

These US, UK, and international programs will have an important public health impact in terms of reducing the NTD and malaria disease burdens in the three Ebola-affected West African countries. A high priority will be to ensure that these life-saving initiatives continue while combating the Ebola epidemic. In November 2014, the White House announced it will seek US$6.18 billion in emergency funds to fight Ebola in West Africa and at home in the US [23]. The request includes large-scale funds for the Centers for Disease Control and Prevention and USAID, but also approximately US$250 million for the National Institutes of Health and Food and Drug Administration to support advanced product and clinical development for new Ebola vaccines and therapeutics. It is also important to highlight the opportunities for integrating control programs for NTDs, malaria, and Ebola, which geographically overlap among affected populations in the three West African countries, as well as the overarching role of strengthening health systems to ensure that funds committed for these diseases are used to their fullest potential. In some cases, the control programs for NTDs, malaria, and Ebola themselves may strengthen health systems, but dedicated independent support and commitment for health systems will also be required.

Finally, it’s essential to be mindful of the need to also support advanced product development for the most common NTDs affecting West Africa. New technologies, including drugs, diagnostics, and vaccines will be needed to eliminate the leading NTDs, and yet, funds are mostly not available for this purpose. Previously, I advocated for allocating 1%–2% of total US global health support—approximately US$100–US$200 million—for such product development [24]. Of note, USAID recently provided US$10 million to Drugs for Neglected Diseases Initiative (DNDi) for new drugs to combat human filarial infections—lymphatic filariasis and onchocerciasis [25]—a trend that could continue for all the major NTDs in order to ensure success in elimination efforts for sub-Saharan Africa and globally. Indeed, a survey of almost 400 NTD experts found that meeting 2012 London Declaration and World Health Assembly resolution targets will not be achieved through mass treatments exclusively [26]. Instead, new technologies may be required. Beyond support from the US government, new international mechanisms are needed to ensure adequate support for such technologies [27].

A massive global response is now underway in the Ebola-affected countries of West Africa. Success on this front may depend on simultaneously fighting Ebola virus infection, NTDs, malaria, and other public health threats.
